# The Impact of Parental Mathematical Education Anxiety and Positive Suggestion Intervention on Children’s Mathematics Achievement

**DOI:** 10.3390/bs16010077

**Published:** 2026-01-06

**Authors:** Dandan Zhou, Boyang Zheng, Yirui Chen, Shasha Yuan, Fang Zhang, Kemeng Qu, Yongxin Li

**Affiliations:** 1 Institute of Psychology and Behavior, Henan University, Kaifeng 475000, China; 2 Kaifeng One Division Attached Elementary School, Kaifeng 475000, China; 3 Pingdingshan Hengshui Zhuoyue Senior High School, Pingdingshan 467000, China

**Keywords:** mathematics achievement, mathematics education anxiety, mathematics learning difficulties, positive suggestion, intervention study

## Abstract

Parental educational anxiety poses a significant risk to children’s academic development. This two-stage study first establishes Parental Mathematics Education Anxiety (PMEA) as a unique construct and then examines the complex effects of a positive suggestion intervention. Study 1, a questionnaire-based investigation, revealed that PMEA is a significant and independent negative predictor of children’s mathematics achievement, distinct from parents’ general state anxiety or their own mathematics anxiety. It also identified socioeconomic factors, such as family income and parental education, as key drivers of PMEA. Study 2 employed an experimental design to test an intervention, revealing that the effectiveness of positive suggestions is not universal but is significantly moderated by the three-way interaction of PMEA level, child type (with/without math learning difficulties), and suggestion frequency. Notably, for non-math-difficult children, frequent positive suggestions from high-anxiety parents were found to be potentially detrimental (a “backfire effect”), whereas for math-difficult children in high-anxiety homes, a higher frequency of suggestion was necessary to yield benefits. These findings deepen the understanding of PMEA’s mechanisms and underscore the necessity of moving beyond one-size-fits-all approaches toward differentiated, context-aware intervention strategies in family education.

## 1. Introduction

The family, as the primary environment for children’s growth, plays an irreplaceable role in their cognitive, emotional, and social development (L. Y. Deng et al., 2023; Gertler et al., 2014; J. C. Liu & Zhao, 2024; Malti & Dys, 2018). In the context of globalized economic competition, a paradigm of ‘intensive parenting’ has emerged, wherein parents, particularly from the middle class, are expected to heavily invest in their children’s educational success to secure their future (Lareau, 2011; Hays, 1996). This global trend creates a high-pressure family environment where parents become acutely sensitive to their children’s academic performance. This phenomenon is particularly pronounced in emerging economies and the Global South, where education is often perceived as the primary vehicle for upward social mobility. In this context, both deviations in parental educational expectations and the high uncertainty in the educational process are significant factors contributing to widespread parental educational anxiety (H. Z. Chen & Xiao, 2014; Yin et al., 2022). This anxiety manifests as excessive worry about children’s academic performance and future development, keeping parents in a state of prolonged tension and unease (H. Z. Chen & Xiao, 2014). This phenomenon of parental educational anxiety has gradually evolved into a collective emotional distress, with parents’ excessively high educational expectations being considered one of its most direct driving factors (Fu et al., 2023). Micro-level studies consistently show that parental anxiety can be transmitted intergenerationally, increasing children’s emotional distress and academic pressure, thereby negatively affecting their academic performance (L. Y. Deng et al., 2023; L. Zhao et al., 2022). Particularly in the field of mathematics, research has found that children of highly anxious parents often exhibit problems such as lack of motivation, passive learning styles, and poor study habits (M. Q. Zhou & Zhou, 2023). Therefore, a deep analysis of the mechanisms by which parental educational anxiety affects children’s learning, and an active search for effective intervention strategies, are of great practical significance for promoting children’s holistic healthy growth and sustainable academic development.

Mathematics, as a foundational discipline, is crucial for the development of children’s abstract thinking, logical reasoning, and problem-solving abilities (Tang, 2024; H. Y. Deng et al., 2020). However, its inherent abstractness, logical rigor, and the complexity of the learning process also pose significant challenges for many children (X. B. Chen, 2007). In the process of tutoring children in mathematics at home, the unpredictability of educational outcomes often leads parents to generate negative self-evaluations and undesirable emotional experiences, thereby triggering specific worries and anxieties about their children’s current mathematics learning status and their own mathematics education abilities, which is termed mathematics education anxiety (Yang, 2022). Although existing research has widely focused on various causes of parental educational anxiety, such as student academic performance, school quality, and family income (Yin et al., 2022; Si & Wang, 2021; L. Li & Yuan, 2019), there has been relatively insufficient exploration of how parental educational anxiety specifically affects children’s learning processes and outcomes. Furthermore, in both practical and theoretical terms, this concept is often confused with the broader concept of “mathematics anxiety”.

Mathematics anxiety is generally defined as the negative emotional experiences such as unease, tension, and fear that an individual experiences when encountering mathematical problems or situations (Ashcraft & Moore, 2009). Research indicates that parental mathematics anxiety can indeed induce mathematics anxiety in children (Carkoglu et al., 2023), which in turn negatively affects children’s engagement and confidence in mathematics (Guan et al., 2020; Cui et al., 2011). This intergenerational transmission of anxiety means that parents’ negative attitudes and beliefs about mathematics can influence children’s cognition and emotions towards mathematics through daily interactions (e.g., tutoring methods, verbal suggestions), thereby exacerbating their levels of mathematics anxiety (X. M. Zhao et al., 2021; T. Zhou & Yi, 2016). However, despite these studies revealing the potential impact of parental emotions on children’s learning, a systematic and in-depth investigation into the precise definition of PMEA as a specific concept and its unique mechanisms affecting children’s mathematics achievement is still lacking, which constitutes a significant theoretical gap in this research.

To clarify the conceptual framework, it is crucial to first provide an operational definition for PMEA and systematically differentiate it from the more established concept of Parental Mathematics Anxiety (PMA). This study defines PMEA as a situation-specific anxiety experienced by parents, which is directly triggered by the context of their child’s mathematics education. This anxiety manifests across several interconnected domains: persistent worries about the child’s current learning status (e.g., poor performance, problematic study habits) and future academic or career prospects; negative emotional reactions such as frustration, anger, and helplessness during tutoring interactions; and a diminished sense of self-efficacy regarding their ability to effectively help their child, often intensified by social comparison. This construct is distinct from PMA in three fundamental ways. First, their sources differ. PMA stems from a parent’s personal history and direct engagement with mathematics (Ashcraft & Moore, 2009), whereas PMEA is vicarious, arising from the parent’s observation of and involvement in their child’s mathematical struggles. Second, their natures differ. PMA is primarily an ability-based anxiety related to one’s own skills. PMEA, in contrast, is an education- and performance-based anxiety, deeply embedded in the modern context of “intensive parenting” and societal pressures for academic success (T. Liu, 2023). A parent may be perfectly comfortable with their own math skills but experience intense PMEA when their child fails to meet expectations. Third, they differ in context-dependency. While PMA can be a stable trait, PMEA is acutely activated by specific situations, such as helping with homework or attending a parent-teacher conference. This distinction is vital because, as the study posits, the anxiety transmitted to a child may not stem from the parent’s abstract fear of math, but rather from the palpable tension present in these direct educational interactions (Elliot & Thrash, 2004; Maloney et al., 2015).

Therefore, PMEA may serve as a more proximal and potent mechanism affecting children’s learning than PMA. It is the immediate, situational anxiety experienced during tutoring that is most likely to be transmitted to the child. Previous studies exploring the relationship between parents and children’s mathematics achievement may have inadvertently conflated these two concepts. For example, studies by Berkowitz et al. (2015) and Schaeffer et al. (2018) found that parental mathematics anxiety affects children’s mathematics achievement through the frequency of homework help. However, from the perspective of their research context and results, these studies actually revealed more about the anxiety generated by parents in specific situations during the process of tutoring their children in mathematics due to the uncertainty of educational outcomes (e.g., slow comprehension by the child, poor grades), which is a typical manifestation of PMEA.

Given the potential negative impact of PMEA on children’s mathematics achievement, early and targeted intervention appears particularly urgent (X. Zhou et al., 2013). Currently, interventions aimed at improving mathematics achievement mostly focus on training children’s own cognitive abilities, such as improving attention and working memory through executive function training (S. K. Li, 2022; Kang & Zeng, 2018), or strengthening their perceptual understanding of quantity and operations through number sense training (C. Y. Wang & Liu, 2013). However, existing research has paid less attention to interventions within the family educational environment, especially for parents who already experience educational anxiety. Suggestion, as a subtle and clever way to influence others’ psychology and behavior, is widely recognized in social psychology (Writing Group of Social Psychology from 13 Universities Nationwide, 1995) and plays an important guiding role in children’s growth (S. K. Li, 2022). Research generally shows that the younger the child, the stronger their suggestibility (L. H. Zhou & Liu, 2003); at the same time, parents, as the most authoritative figures in children’s growth, can play a particularly significant role in suggestion interventions (Lu, 2014; Zhan et al., 2001). Furthermore, previous research has clearly pointed out that parents with different levels of PMEA exhibit significant differences in their educational behaviors. Highly anxious parents may be overly concerned with grades and tend to adopt suboptimal parenting styles, while less anxious parents show higher educational involvement and emotional engagement (M. Q. Zhou & Zhou, 2023). These findings strongly suggest that intervention programs designed for different levels of PMEA may have significantly different effects, implying that, in practice, parental anxiety levels need to be considered to achieve more precise and efficient intervention outcomes. Therefore, the question is whether the effectiveness of interventions like positive suggestion varies across different levels of family PMEA. Answering this question is a crucial step toward moving from one-size-fits-all interventions to personalized guidance.

This study also focuses on children with mathematics learning difficulties (hereinafter referred to as math-difficult children/MD children), a special group who exhibit significant delays in mathematics learning (L. Chen & Zhao, 2013), with an incidence rate of about 4% to 7% (Fuchs et al., 2005). Existing research mostly focuses on exploring the cognitive ability deficits of MD children themselves (Geary et al., 2012; Geary et al., 2007), such as their deficiencies in word problem-solving ability (Y. Wang et al., 2022) or self-monitoring ability (Han & Zhao, 2020), but the attention to external environmental factors, especially parental influence, is still insufficient. Given the uniqueness and vulnerability of MD children, it is of great significance to deeply explore the specific impact of PMEA on them and the effectiveness of positive suggestion interventions.

This situational anxiety is further exacerbated by the global phenomenon of “shadow education”—the vast system of private tutoring that parallels mainstream schooling (Kim & Jung, 2019). In many countries across the Global South, from India to Nigeria, reliance on private tutoring is not a choice but a perceived necessity to compensate for shortcomings in the public education system (Bray & Kwo, 2014). The financial and emotional investment in this parallel system makes parents hyper-vigilant about educational outcomes, a key trigger for PMEA. PMEA is a highly situational anxiety, the emergence of which may stem directly from children’s mathematics performance not meeting parental expectations or from being overly influenced by external educational propaganda (e.g., “chicken baby” culture, pressure for higher education) (Zhu & Luo, 2023; Yin et al., 2022). Given the significant intergenerational transmissibility of anxiety (Carkoglu et al., 2023) and the crucial role of parents as core influencers in children’s learning environments (T. Liu, 2023; Cosso et al., 2022), the potential negative impact of PMEA on children’s learning cannot be overlooked. Therefore, this study believes that clearly distinguishing between these two concepts is crucial for accurately identifying problems in family education and formulating more targeted and effective intervention strategies. While established instruments assess students’ test/school anxiety and adults’ mathematics anxiety, there remains a measurement gap for parents’ situational educational anxiety in home tutoring contexts. The present PMEA instrument is designed to address this gap and to advance research on family-based mechanisms affecting children’s mathematics achievement.

In summary, this study aims to fill the existing gaps in current research regarding the precise distinction of the concept of PMEA, its unique mechanisms affecting children’s mathematics learning, and family education intervention strategies. To achieve this, the study seeks to address the following core questions and test corresponding hypotheses: (1) Does PMEA independently and negatively affect children’s mathematics achievement, separate from parents’ state anxiety and mathematics anxiety? (2) Is the enhancing effect of positive suggestion intervention on children’s mathematics achievement moderated by PMEA levels and child types (MD children/non- math-difficult children, hereinafter referred to as NMD children)?

To systematically answer these questions, this study adopts a closely integrated two-stage research design that progresses from problem identification to solution testing. The entire design is built on a strategic foundation: Study 1, a questionnaire-based investigation, not only establishes the uniqueness of PMEA as an independent predictor but also serves a crucial preparatory role for the experiment. The use of purposive sampling in Study 1 (i.e., selecting MD and NMD children) was a deliberate choice designed to: (1) enhance statistical power by increasing the variance in the dependent variable (mathematics achievement) to more effectively test the association between PMEA and achievement; (2) focus the theoretical test by ensuring a sufficient MD sample to validate the core hypothesis; and (3) establish an experimental foundation by pre-establishing the critical participant groups for the moderation analysis in Study 2. This transforms Study 1 from a simple correlational survey into a strategic foundation for the subsequent experimental phase. Building upon this foundation, Study 2 then employs an experimental design to precisely test the moderating effects of PMEA level and child type on the intervention’s effectiveness, facilitating a deep exploration from identifying the problem to validating a differentiated solution.

This research aims to provide new perspectives and differentiated intervention strategies for alleviating PMEA and improving children’s mathematics learning performance, and to offer valuable practical insights for building a harmonious and effective home-school collaborative education model. The present exploratory study prioritizes theory-driven item development, content validity, and internal consistency for the PMEA scale; comprehensive structural validation (EFA/CFA, measurement invariance, convergent/discriminant and cross-cultural validation) is planned for subsequent work. This study believes that clearly distinguishing between these two concepts is crucial for accurately identifying problems in family education and formulating more targeted and effective intervention strategies. While established instruments assess students’ test/school anxiety and adults’ mathematics anxiety, there remains a measurement gap for parents’ situational educational anxiety in home tutoring contexts. The present PMEA instrument is designed to address this gap and to advance research on family-based mechanisms affecting children’s mathematics achievement.

## 2. Study 1: The Impact of PMEA on Children’s Mathematics Achievement

### 2.1. Research Objectives and Participants

This study aims to explore the intrinsic relationship between parental state anxiety, mathematics anxiety, PMEA, and children’s mathematics achievement. Third-grade students and their parents were selected as participants, based on two key considerations: the third grade is a critical period for academic development where mathematics performance often shows clear differentiation, and concurrently, it is a stage where parents commonly exhibit significant worry and anxiety regarding their children’s academic progress (Yang, 2022; X. G. Wang, 2017).

The data were collected in mid-December 2020 from a public primary school in Kaifeng, Henan Province, China, which had approximately 800 third-grade students. A multi-stage screening process was employed to select participants. First, to identify students with potential mathematical difficulties, all students were ranked based on the average scores of their two most recent major exams. Those whose mathematics ranking was in the bottom 30% were initially selected. To exclude general learning difficulties, only students with Chinese language scores at or above a moderate level were retained. Next, using a clinical diagnostic method, class teachers and mathematics teachers confirmed that these selected students had no obvious sensory impairments (e.g., visual or auditory impairments) or emotional disorders (e.g., depression). This process yielded the math-difficult (MD) group consisting of 143 students. To ensure comparability, a non-math-difficult (NMD) group of 138 students was then randomly sampled from the remaining students, matched at the class and gender level.

Questionnaires were subsequently distributed to the parents of all selected students. A total of 281 questionnaires were sent out, and 269 were returned. After excluding 9 incompletely filled questionnaires, the final valid sample consisted of 260 student-parent pairs. The sample was composed of 134 students in the MD group (68 boys and 66 girls) and 126 students in the NMD group (58 boys and 68 girls). In accordance with the diagnostic criteria for Specific Learning Disorder outlined in the DSM-5 (American Psychiatric Association, 2013), it is essential to rule out intellectual disability as the primary cause of academic deficits. Therefore, as a final validation step, students in the MD group completed Raven’s Standard Progressive Matrices (Raven, 1941; H. C. Zhang & Wang, 1989). All students scored above the 5th percentile, a commonly used threshold in research to exclude intellectual deficits (Fuchs et al., 2005), thereby validating the specificity of their mathematical difficulties. The Cronbach’s α coefficient for this test in the study was 0.92, indicating good reliability.

This phase’s sampling was not only aimed at exploring the general relationship between PMEA and mathematics achievement; its stratified screening strategy (i.e., pre-distinguishing MD and NMD children) was also foundational to the experimental design of Study 2, ensuring the precision of the subsequent intervention effect analysis. All participants provided informed consent and voluntarily participated. This study received ethical approval and was conducted following the principles of the Declaration of Helsinki. Please see the Institutional Review Board Statement for a detailed account of the ethical considerations. Throughout the screening process, the research team emphasized to all participants that this grouping was for research purposes only and did not represent a definitive judgment of a student’s abilities. All data were strictly anonymized to prevent any potential labeling effects or psychological distress.

### 2.2. Research Instruments

#### 2.2.1. Demographic and Achievement Data

Data were collected via a demographic information sheet completed by the parent who was primarily responsible for tutoring the child in mathematics. This sheet included items on the parents’ role (father/mother), the child’s gender, single-child status, family structure (two-parent/non-two-parent), parental education level, and family monthly income.

Children’s mathematics achievement was measured by the average of their scores from the two most recent major examinations of the semester, obtained from official school records. These standardized examinations, organized by the municipal education authority and administered uniformly across all public schools in the district, ensure the comparability of the scores. The rationale for using these particular scores over a general external test was to maximize ecological validity. Given that PMEA is a direct response to a child’s performance within their actual academic context, these city-wide unified exam scores serve as the most authentic and relevant outcome measure for this study.

#### 2.2.2. State Anxiety Inventory (SAI)

Developed by Spielberger et al. (1971) and validated for reliability and validity by X. H. Zheng et al. (1993), this self-report questionnaire comprises 20 items with a 4-point Likert scale. Higher total scores indicate higher levels of anxiety. In this study, the Cronbach’s α coefficient for this scale was 0.91, indicating good internal consistency reliability.

#### 2.2.3. Mathematics Anxiety Scale

This scale, developed by Plake and Parker (1982) and revised by X. Z. Liu (2009), contains 21 items with a 5-point Likert scale. Higher average scores on all items indicate higher levels of mathematics anxiety. The Cronbach’s α coefficient for the scale was 0.96, demonstrating good internal consistency reliability.

#### 2.2.4. Parental Mathematics Education Anxiety Questionnaire

Given that existing questionnaires in the literature primarily measure parental mathematics anxiety—anxiety related to the parents’ own mathematical abilities—a dedicated instrument for assessing the situational PMEA that arises specifically from tutoring one’s child was lacking. To fill this measurement gap, the PMEA questionnaire was developed. It was designed to comprehensively assess situational anxiety among parents in the context of their children’s math education. Its construction was theory-driven, encompassing four predefined dimensions used for item generation: (1) the child’s mathematics learning status, (2) the child’s future development, (3) parental mathematics tutoring, and (4) parents’ self-perception. An initial pool of 29 items was generated and subsequently evaluated for content validity by 49 psychology graduate students. Following this assessment, 8 items with an item-level Content Validity Index (I-CVI) below 0.78 were removed, resulting in a final 21-item instrument. The questionnaire employs a 5-point Likert scale, with higher total scores indicating greater levels of PMEA; it includes reverse-keyed items that were flagged and scored accordingly. In this study, the overall instrument demonstrated good internal consistency, with a Cronbach’s α coefficient of 0.87. It should be noted that this questionnaire is in its initial stage of development. While its content validity was assessed, a full validation of its construct validity (e.g., factor analysis) is a key direction for future research. The findings of the current study should, therefore, be interpreted in this context. The full questionnaire, including dimension mapping and reverse-key annotations, is provided in Appendix A to facilitate reproducibility.

### 2.3. Results and Discussion

#### 2.3.1. Differential Analysis of Demographic Variables

First, a descriptive statistical analysis of the demographic variables was conducted. The results are presented in Table 1. Due to a significant sample imbalance in three variables—Parental Role, Single-Child Status, and Family Structure—where the minority categories accounted for less than 13% of the sample, these variables were not included in subsequent inferential statistical analyses to ensure statistical validity.

A series of two-tailed independent samples *t*-tests was conducted to investigate whether significant differences existed in parental state anxiety, mathematics anxiety, and PMEA based on child’s gender, parental education, and monthly family income. The family income variable was dichotomized using a 5000 RMB threshold. This was justified on two grounds: it serves as a common socioeconomic benchmark for the region, and it effectively split the sample into two comparably sized groups (*n* = 125 vs. *n* = 135), confirming its suitability as a median split.

A series of independent samples *t*-tests was conducted to examine the influence of demographic variables on parental anxiety. The means, standard deviations, and full results are presented in Table 2. Regarding child’s gender, no statistically significant differences were found in parents’ state anxiety, *t*(258) = −0.246, *p* = 0.806, Cohen’s *d* = −0.030, or their mathematics education anxiety, *t*(258) = −1.663, *p* = 0.098, Cohen’s *d* = −0.206. However, parental mathematics anxiety was significantly higher for parents of girls than for parents of boys, *t*(258) = −2.503, *p* = 0.013, Cohen’s *d* = −0.311. Turning to socioeconomic factors, monthly family income showed no significant effect on state anxiety, *t*(258) = 1.626, *p* = 0.105, Cohen’s *d* = 0.202, but had a significant impact on both mathematics anxiety, *t*(258) = 2.094, *p* = 0.037, Cohen’s *d* = 0.260, and mathematics education anxiety, *t*(258) = 3.041, *p* = 0.003, Cohen’s *d* = 0.374. Specifically, parents with a monthly income below 5000 RMB exhibited higher levels of both anxiety types. Similarly, parental education level was also a key factor. Parents with lower educational attainment reported significantly higher state anxiety, *t*(258) = −2.10, *p* = 0.037, Cohen’s *d* = 0.310, and mathematics education anxiety, *t*(258) = 2.584, *p* = 0.010, Cohen’s *d* = 0.329. Their mathematics anxiety also approached marginal significance, *t*(258) = 1.811, *p* = 0.071, Cohen’s *d* = 0.230. A subsequent correlation analysis confirmed that parental education level was significantly and positively correlated with monthly family income, *r* = 0.315, *p* < 0.001, suggesting an interconnection between these socioeconomic factors.

#### 2.3.2. Descriptive Statistics and Correlation Analysis

Descriptive statistics and a correlational analysis were conducted to examine the relationships among parental state anxiety, mathematics anxiety, PMEA, and children’s mathematics achievement (see Table 3). The results indicated significant positive correlations among all three types of parental anxiety, which aligns with existing findings on the general comorbidity of anxiety emotions. However, when examining their relationship with children’s mathematics achievement, only parental mathematics anxiety (*r* = −0.194, *p* = 0.002) and mathematics education anxiety (*r* = −0.333, *p* < 0.001) were significantly and negatively correlated with children’s mathematics achievement. This key finding reveals that higher levels of parental math-related anxieties are associated with poorer mathematics performance in their children.

#### 2.3.3. Multiple Linear Regression Analysis

A multiple linear regression analysis was conducted to examine the independent predictive effects of parental state anxiety, mathematics anxiety, and mathematics education anxiety on children’s mathematics achievement (see Table 4). The overall model was statistically significant, *F* (3,256) = 11.213, *p* < 0.001, and accounted for approximately 11.6% of the variance in children’s mathematics achievement (R^2^ = 0.116). The detailed results are presented in Table 4. After controlling for both parental state anxiety and mathematics anxiety, PMEA remained the only significant negative predictor of children’s mathematics achievement [*β* = −0.303, *t*(258) = −4.746, *p* < 0.001]. Neither parental state anxiety [*β* = −0.004, *t*(258) = −0.063, *p* = 0.950] nor parental mathematics anxiety [*β* = 0.075, *t*(258) = −1.138, *p* = 0.256] were significant predictors in the model. These results indicate that PMEA level has a unique and robust association with children’s mathematics achievement, independent of other forms of parental anxiety.

#### 2.3.4. Discussion

This result lends support to the hypothesis that PMEA is an important factor affecting children’s mathematics achievement, and the effect is not simply attributed to general parental state anxiety or their own mathematics anxiety. However, it is important to note that this correlational finding does not establish causality. Given that the child’s learning status is a core dimension of the PMEA construct, it is plausible that the relationship is bidirectional, where a child’s struggles may, in turn, elevate parental anxiety. While the relationship is likely bidirectional, the findings are nonetheless consistent with the parent-to-child effect proposed by theoretical models of intergenerational transmission (Chorpita & Barlow, 1998). The mechanisms of this effect are likely multifaceted. Some literature suggests that highly anxious parents are more likely to adopt controlling parenting styles, such as excessive intervention and displaying impatience, which in turn undermine children’s learning autonomy and create negative emotional experiences (Oh et al., 2022; Retanal et al., 2021). For example, M. Q. Zhou and Zhou (2023) found that highly anxious parents were more likely to adopt controlling tutoring styles, which correlated with lower learning quality in students. Similarly, L. Y. Deng et al. (2023) identified that parental anxiety could be transmitted to children, increasing their academic stress. Furthermore, these negative parental messages and behaviors can be internalized by children, lowering their mathematics self-efficacy and causing them to doubt their own abilities (Carkoglu et al., 2023).

The findings of Study 1 not only confirm the unique negative impact of PMEA on children’s mathematics achievement but, more importantly, they highlight two variables critical for future interventions: PMEA level and child type (MD vs. NMD). These findings suggest that the effects of any intervention are likely not universal but highly contextual. Would a seemingly beneficial intervention (like positive suggestion) produce significantly different outcomes across these four distinct quadrants (High/Low PMEA × MD/NMD children)? To move from correlational findings to causal testing and to explore differentiated intervention strategies, the Study 2 was designed to directly answer this question using an experimental approach.

## 3. Study 2: Research on the Enhancing Effect of Positive Suggestion from PMEA on Children’s Mathematics Achievement

### 3.1. Research Objectives and Participants

Building on the critical impact of PMEA and its variance across different child groups established in Study 1, Study 2 was designed to experimentally test the effectiveness of a positive suggestion intervention and to deeply examine whether its effects are moderated by parental PMEA level and child type. Participants for this experimental phase were selected from the original cohort of 260 student-parent pairs from Study 1 using a stratified sampling strategy based on two criteria: child type and PMEA.

First, parents from the Study 1 sample were categorized based on PMEA scores. Those whose scores fell in the top 30% were assigned to the “High Anxiety Group”, while those in the bottom 30% were assigned to the “Low Anxiety Group”. To validate this grouping, independent samples *t*-tests were conducted, which confirmed a significant difference in PMEA scores between the high and low anxiety groups for both MD children, *t*(58) = −18.204, *p* < 0.001, Cohen’s *d* = −4.700, and NMD children, *t*(58) = −18.826, *p* < 0.001, Cohen’s *d* = −4.861. This procedure yielded four validated subgroups: (1) MD children with high-anxiety parents, (2) MD children with low-anxiety parents, (3) NMD children with high-anxiety parents, and (4) NMD children with low-anxiety parents. From each of these four subgroups, 30 student-parent pairs were randomly selected. This sample size (*n* = 30 per group) is consistent with conventions in experimental educational psychology research, which often recommends a minimum of 20–30 participants per cell to ensure adequate power for detecting meaningful effects (e.g., Cohen, 1992; Simmons et al., 2011). This resulted in a final sample of 120 pairs for Study 2, ensuring an equal and robust distribution of participants across the four experimental conditions (MD/High-Anxiety, MD/Low-Anxiety, NMD/High-Anxiety, NMD/Low-Anxiety).

### 3.2. Positive Suggestion Intervention Check-In Questionnaire

To quantify intervention frequency and validate the experimental manipulation, a self-developed “Positive Suggestion Intervention Check-in Tool” was utilized. It is crucial to note that this was not a dependent measure but a logistical tool used to record whether parents had implemented the intervention each day. The data derived from this tool—the total number of days each parent completed the check-in—was subsequently used to operationalize the “Suggestion Intervention Frequency” variable for analysis.

The development of this tool was rigorous. First, an initial pool of 28 items was generated to provide concrete examples of supportive behaviors, grouped into three practical categories for parents: language (e.g., verbal praise), body movements (e.g., a thumbs-up), and facial expressions (e.g., a warm smile). These items were then evaluated for content validity by 49 psychology graduate students. Following their feedback, 7 items with an item-level Content Validity Index (I-CVI) below 0.78 were removed. The final instrument thus comprised 21 distinct positive suggestion items: 8 for language, 8 for body movements, and 5 for facial expressions.

### 3.3. Experimental Procedure

This study followed a pre-test-intervention-post-test design. The effectiveness of the intervention was evaluated by analyzing the participants’ post-test mathematics achievement scores as the primary outcome, after first using pre-test scores to validate the baseline characteristics of the experimental groups. The procedure consisted of the following stages:

Parent Training: Prior to the intervention, a single online guidance session was conducted for all participating parents. During this session, the research objectives were explained, and parents were instructed on how to implement positive suggestions (e.g., verbal encouragement, supportive gestures, and physical contact) during daily mathematics tutoring. Parents were also trained on how to use the check-in questionnaire to confirm their daily participation. The training did not cover any specific mathematics content.

Pre-test: The pre-test data consisted of the students’ average mathematics scores from the two school-wide joint examinations that were administered before the intervention period began. This data was used to validate the baseline differences between the MD and NMD groups and the equivalence between the anxiety groups.

Intervention and Quantification of Frequency: The intervention phase lasted for a total of 32 days for all participants. This timeframe was strategically determined by the academic calendar, fitting precisely between two consecutive city-wide unified examinations, thereby maximizing the ecological validity of the pre-test and post-test measures. During this period, parents were required to implement the positive suggestion strategies during their daily tutoring sessions and, after each session, promptly fill in the check-in questionnaire to record that an intervention had occurred that day. To quantify the intervention intensity, the total number of days each parent completed the check-in during this 32-day period was recorded. A distribution analysis revealed that the intervention frequency was significantly non-normal (Shapiro–Wilk *W* = 0.868, *p* < 0.001) and exhibited a clear bimodal distribution (Bimodality Coefficient *BC* = 0.653). This bimodal structure suggests the presence of two distinct subgroups of participant engagement. Further supporting this, the center of the distribution was sparsely populated; only 34 participants (28.3%) had intervention frequencies between 9 and 19 days. Given this data structure, dichotomizing the variable is not merely a statistical convenience but a data-driven approach to capture these two natural behavioral clusters. The median value of 13.5 falls precisely within a “frequency valley,” with only 6 individuals (5% of the sample) recorded at exactly 13 or 14 days. This positioning confirms that the median split does not divide a dense, homogeneous group but instead serves as a meaningful cut-off point that distinguishes two distinct behavioral patterns. This justifies the use of a dichotomous frequency variable for the subsequent factorial ANOVA. Consequently, to prepare the data for this analysis, parents who checked in on 14 or more days (above the median) were classified as the “High Suggestion Group,” while those who checked in on fewer than 14 days (below the median) were classified as the “Low Suggestion Group.” This standard, data-driven procedure ensured an objective division of the sample for the “Suggestion Intervention Frequency” variable.

Post-test: The post-test data was the students’ official mathematics scores from the final examination, which was administered on the day immediately following the conclusion of the 32-day intervention. These scores served as the primary dependent variable for the main analysis.

To ensure comparability across these assessments and control for variations in test difficulty, all mathematics scores were standardized by converting them into T-scores (*M* = 50, *SD* = 10). Consequently, the post-intervention T-score served as the primary dependent variable for evaluating the intervention’s effectiveness, while the pre-intervention T-score was used to validate the baseline characteristics of the experimental groups. The means and standard deviations for the pre- and post-intervention mathematics achievement T-scores for all experimental groups are presented in Table 5.

### 3.4. Results and Discussion

#### 3.4.1. Baseline Validation Using Pre-Intervention Achievement Scores

**Figure 1:**
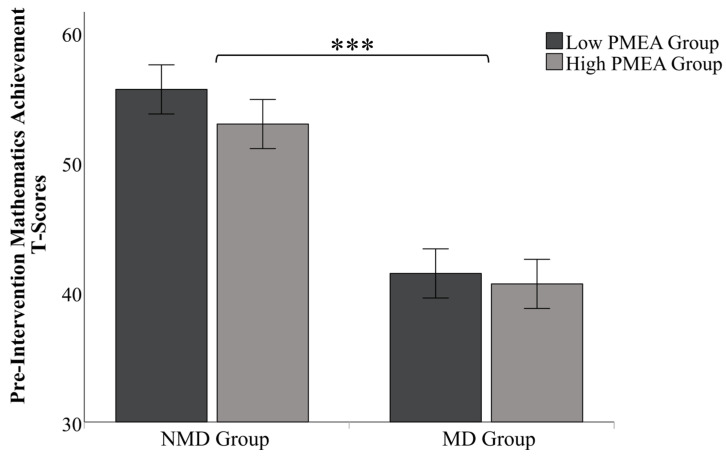
Differences in children’s mathematics achievement before positive suggestion intervention across different variables. Note: *** *p* < 0.001.

To validate the experimental groups, a 2 (child type: MD, NMD) × 2 (PMEA level: low, high) ANOVA was conducted on the pre-intervention T-score (see Figure 1). The analysis confirmed a significant main effect for child type [*F* (1,116) = 192.240, *p* < 0.001, η_p_^2^ = 0.624], with NMD group children (*M* = 54.28, *SD* = 4.57) scoring significantly higher than MD group (*M* = 41.04, *SD* = 5.91). This finding validates the operational definition of the child type classification. Crucially, the main effect for PMEA level, while not significant at the α = 0.05 threshold, was found to be marginally significant, [*F* (1,116) = 3.305, *p* = 0.07, η_p_^2^ = 0.03]. This suggests a potential pre-existing difference in mathematics achievement between children of low- and high-anxiety parents. The interaction effect between child type and PMEA level was not significant, [*F* (1,116) = 0.946, *p* = 0.333, η_p_^2^ = 0.008]. This lack of interaction demonstrates that there were no significant differences in baseline achievement between children of high- and low-anxiety parents within either the MD group or the NMD group. Despite the absence of significant differences within each child type subgroup, the overall marginal significance of the PMEA main effect warranted a cautious approach. Therefore, to control for any potential confounding influence of baseline scores, it was deemed necessary to include the pre-intervention T-score as a covariate in the subsequent analysis of intervention effects.

#### 3.4.2. Analysis of Intervention Effects Using Post-Intervention Achievement Scores

To determine the intervention’s effect, this study employed an Analysis of Covariance (ANCOVA) within a 2 (child type: MD, NMD) × 2 (PMEA level: low, high) × 2 (suggestion frequency: low, high) factorial design framework. This approach was chosen as it is a classic and direct statistical method for testing interaction effects (i.e., moderation) in experimental designs. While mathematically equivalent to testing interaction terms in a regression framework, it offers a clearer presentation of mean differences across experimental conditions. Given the marginal significance of the PMEA main effect on pre-intervention T-scores, the pre-intervention T-score was included as a covariate to control for baseline differences, with the post-intervention T-score serving as the dependent variable.

The results confirmed that the covariate, pre-intervention achievement, was significant [*F* (1,111) = 13.039, *p* < 0.001, η_p_^2^ = 0.105], indicating that controlling for baseline scores was appropriate. After this adjustment, there was a significant main effect for PMEA level, [*F* (1,111) = 6.587, *p* = 0.012, η_p_^2^ = 0.056], showing that children from low-PMEA families achieved significantly higher post-intervention scores than those from high-PMEA families. The main effects for child type, [*F* (1,111) = 0.732, *p* = 0.394, η_p_^2^ = 0.007], and suggestion frequency, [*F* (1,111) = 1.255, *p* = 0.265, η_p_^2^ = 0.011], were not significant. All two-way interactions were also not significant (all *ps* > 0.28). Most critically, the analysis revealed a significant three-way interaction among child type, PMEA level, and suggestion frequency, [*F* (1,111) = 5.322, *p* = 0.023, η_p_^2^ = 0.046].

**Figure 2:**
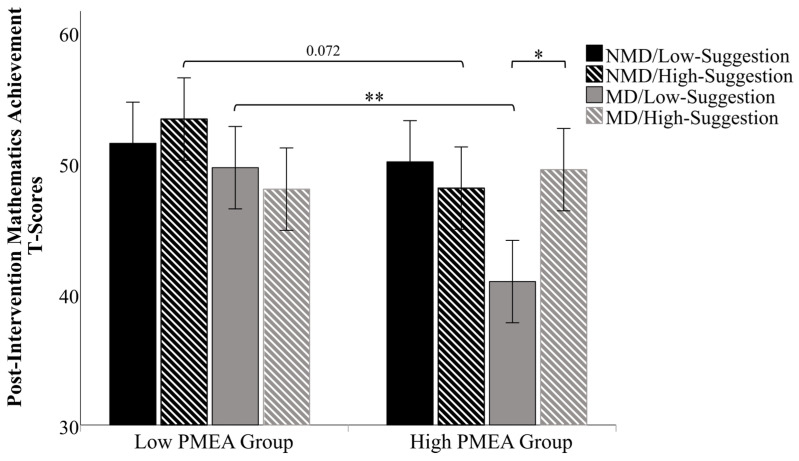
Differences in children’s mathematics achievement after positive suggestion intervention across different variables. Note: * *p* < 0.05, ** *p* < 0.01.

To decompose this complex interaction, simple effects analysis was conducted, revealing a distinct pattern of intervention effectiveness for each child type (see Figure 2). For NMD children, the family’s emotional context appeared to be the dominant factor moderating the intervention’s impact, revealing a potential “backfire effect.” The primary evidence for this backfire effect is a marginally significant trend when comparing anxiety groups under the high-frequency suggestion condition: children from low-PMEA families (*M* = 53.34) significantly outperformed those from high-PMEA families (*M* = 48.07) (*p* = 0.072, η_p_^2^ = 0.029), showing the intervention had opposite effects in the two contexts. While the direct effect of suggestion frequency within the high-PMEA group did not reach statistical significance, its directionality provides a clear explanation for this divergence. Specifically, high-frequency suggestions led to a lower mean score (*M* = 48.07) compared to low-frequency suggestions (*M* = 50.08; *p* = 0.447, η_p_^2^ = 0.005). This trend is opposite to that in low-PMEA families (*p* = 0.433, η_p_^2^ = 0.006). This effect will be interpreted in depth in Section 4 through the lens of Self-Determination Theory.

For MD children, the results showed a clear interactive effect between parental anxiety and intervention frequency. In a low-anxiety (low-PMEA level) environment, children performed well regardless of suggestion frequency; in fact, there was a significant advantage for children from low-PMEA families over high-PMEA families when the suggestion frequency was low (*p* = 0.003, η_p_^2^ = 0.077), suggesting a protective effect of the low-anxiety context itself. Conversely, for MD children in a high-anxiety (high-PMEA) environment, the intervention frequency was critical: high-frequency suggestions led to significantly better mathematics achievement than low-frequency suggestions (*p* = 0.012, η_p_^2^ = 0.056). This indicates that for this vulnerable group, a more intensive intervention is necessary to overcome the negative impact of a high-anxiety home environment.

In summary, the findings of Study 2 clearly indicate that the enhancing effect of positive suggestion intervention on children’s mathematics achievement is not universal, but is significantly moderated by PMEA levels and child types (MD children/NMD children). This provides direct experimental evidence for understanding the boundaries of family intervention effectiveness and emphasizes the necessity of adopting differentiated and individualized strategies in practice.

## 4. General Discussion

The findings from this two-stage study systematically reveal the central role of PMEA in family mathematics education, transitioning logically from correlation to causation. Study 1 first established PMEA as a more potent and independent negative predictor of children’s mathematics achievement than general anxiety or parental math anxiety, providing a critical target for intervention. Critically, it also identified two key variables—PMEA level and child type (MD vs. NMD)—that set the stage for a more nuanced investigation. Subsequently, the experimental results of Study 2 directly tested this, demonstrating that interventions targeting PMEA are not universally effective. Their boundaries are precisely defined by the very variables identified in Study 1. The ‘backfire effect’ observed in high-anxiety parents, in particular, strongly illustrates that an intervention ignoring the family’s initial emotional state may not only be ineffective but potentially harmful. Taken together, the findings from both studies converge on a central conclusion: family mathematics education interventions must move beyond a one-size-fits-all approach toward personalized strategies based on the family’s emotional context and the child’s characteristics. In the following sections discuss the theoretical mechanisms, practical implications, and the socioeconomic drivers behind these key findings in greater detail.

### 4.1. Parental Mathematics Education Anxiety Affects Children’s Mathematics Achievement

As established in the introduction, the first key finding of this research is the critical role of PMEA as an independent predictor of children’s mathematics achievement. This result substantiates the primary hypothesis and empirically distinguishes PMEA as a unique, context-specific construct whose mechanisms warrant a deeper exploration.

Firstly, as core participants in children’s early education, parents’ emotional states directly influence children’s learning experiences (Yu et al., 2025). Highly mathematics-education-anxious parents may unconsciously transmit their anxiety, worry, or even frustration to their children, leading children to associate mathematics achievement with negative emotions (such as tension, pressure, and fear). This negative emotional association significantly reduces children’s motivation and intrinsic interest in mathematics, causing them to develop resistance when facing mathematical tasks (J. Zhang et al., 2018), thereby forming a vicious cycle of ineffective learning. Moreover, the ways in which children’s anxiety is triggered can be multimodal (Q. Zheng & Xu, 2020; H. L. Huang & Zhang, 2019; Rui & Ji, 2017), including both non-verbal cues (such as parents’ facial expressions, changes in tone of voice, and uneasy body language during tutoring) and verbal cues (such as frequent complaints about the difficulty of mathematics, overemphasis on the importance of exams, or doubts about the child’s learning ability).

Secondly, when children perceive PMEA, their own psychological and cognitive resources may be occupied by additional emotional burdens, making it difficult for them to concentrate on mathematics learning tasks themselves (J. Zhang et al., 2018). Research indicates that anxiety affects individuals’ attention allocation and memory processing efficiency (García et al., 2016). When children are chronically in a cognitive state affected by anxiety, their working memory capacity may be limited, and information processing speed may slow down, leading to a decline in their ability to understand mathematical concepts, solve problems, and retain knowledge (Chorpita & Barlow, 1998; Guan et al., 2020; Geng & Chen, 2005). This “cognitive internal consumption” effect significantly weakens children’s ability to handle mathematical problems, making them more prone to frustration and helplessness when facing learning challenges (L. Li & Zhou, 2016).

Furthermore, highly mathematics-education-anxious parents may adopt controlling or oppressive parenting styles. For example, they may excessively intervene in children’s learning processes, frequently conduct tests and evaluations to monitor grades, and overemphasize scores while neglecting the genuine understanding of mathematical concepts and the development of abilities. This excessive control and high-pressure parenting style can lead to strained parent–child relationships and weaken children’s learning autonomy and intrinsic motivation (Retanal et al., 2021). When children feel that learning is forced rather than voluntary, their learning efficiency and effectiveness will be greatly reduced, and they may even develop rebellious psychology, further exacerbating their learning difficulties (Y. J. Chen et al., 2014; S. Huang & Huo, 2014).

Finally, PMEA may also indirectly affect children’s mathematics achievement by influencing children’s mathematics self-efficacy. When parents exhibit mathematics education anxiety, they may transmit negative information and expectations such as “you might not be good at math” or “math is hard, you can’t do it.” These negative messages, internalized by children, will significantly lower their mathematics self-efficacy (Carkoglu et al., 2023; J. Zhang et al., 2018). Children with low mathematics self-efficacy are more likely to withdraw, lack persistence, and believe that their efforts cannot change the outcome when faced with mathematical challenges (P. Li & Cao, 2021; Gao & Chen, 2017), thereby affecting their learning engagement and ultimate achievement.

The findings of this study, as well as the analysis of the mechanisms by which the family emotional environment influences children’s academic performance, provide guidance for future intervention research, specifically that interventions should precisely focus on parents’ specific anxiety about mathematics education, rather than generalized anxiety or merely concerns about parents’ own mathematical abilities.

### 4.2. Positive Suggestion Intervention by Mathematics Education Anxious Parents Improved Children’s Mathematics Performance

The experimental results of Study 2 first reinforce the pervasive impact of PMEA. It is crucial to highlight that even after controlling for initial mathematics performance, a significant main effect persisted: children from low-PMEA families achieved significantly higher post-intervention scores than those from high-PMEA families. This result provides robust experimental evidence that corroborates the correlational findings of Study 1, demonstrating that the negative impact of a high-anxiety home environment is so persistent that it continues to shape academic outcomes even throughout an intervention period.

Moving beyond this now experimentally reinforced impact of PMEA, the second core contribution was to experimentally reveal the complexity and boundary conditions of the positive suggestion intervention. However, it is important to interpret these findings within the context of methodology. The 32-day intervention period, as noted by Lally et al. (2010), is likely insufficient for lasting habit formation. Therefore, the results should be understood as demonstrating powerful short-term effects, while the long-term sustainability of these changes remains a question for future research. With this caveat in mind, the findings clearly show that the intervention is not a universal “panacea.” Its effectiveness is profoundly moderated by both parental anxiety and child type, which has significant guiding implications for developing individualized and precise family education intervention strategies.

The results for non-mathematics learning difficulties children (non-MD children) were particularly revealing, showcasing how the family’s emotional context acts as a critical moderator for the intervention. The key finding is not that positive suggestion is universally effective, but that its outcome depends entirely on the parental anxiety level. This was most evident under the condition of high-frequency suggestions: children from low-anxiety families significantly outperformed those from high-anxiety families, a trend that approached statistical significance (*p* = 0.072). This suggests that a low-anxiety environment is a necessary precondition for intensive positive interventions to be beneficial. For children of high-anxiety parents, the same high-frequency intervention was not merely ineffective but potentially detrimental, leading to the “backfire effect”. This stark contrast highlights that the family’s emotional climate is the primary determinant of whether positive suggestions are perceived as genuine support or as a form of controlling pressure. The backfire effect can be explained more deeply through the lens of Self-Determination Theory (SDT). SDT posits that intrinsic motivation and well-being are contingent on the satisfaction of basic psychological needs for autonomy, competence, and relatedness (Ryan & Deci, 2000). In a high-anxiety home environment, a parent’s frequent “positive suggestions” may fail to be perceived by the child as autonomy-supportive. Instead, they are likely interpreted as a form of controlling behavior. The parents’ underlying anxiety can be transmitted through non-verbal cues, turning the “suggestions” into a form of implicit pressure: “You must perform well to alleviate my anxiety”. This perceived control directly thwarts the child’s need for autonomy, shifting the locus of causality for learning from internal to external. Research consistently shows that when children perceive parenting as controlling rather than autonomy-supportive, their intrinsic motivation, engagement, and ultimately, academic achievement suffer (Grolnick & Ryan, 1989; Oh et al., 2022; Retanal et al., 2021). Therefore, even as a statistical trend, this finding is practically and theoretically meaningful, cautioning that interventions must be considered in light of how they might be interpreted within specific emotional contexts.

For children with mathematics learning difficulties (MD children), the study results presented a more complex and enlightening pattern. Firstly, even a small amount of positive suggestion from low-mathematics education anxiety parents could produce significant positive effects on MD children. This may be due to the fact that low-mathematics education anxiety parents themselves tend to impose less additional emotional pressure or academic burden on MD children (Oh et al., 2022; Retanal et al., 2021; Elliot & Thrash, 2004), and the small amount of positive suggestion they provide can effectively alleviate the intrinsic anxiety and frustration experienced by MD children due to learning difficulties, thereby enhancing their engagement and enthusiasm in mathematics learning. Secondly, for MD children of highly mathematics-educated anxious parents, the study found that more frequent and higher-frequency positive suggestions were required to achieve better intervention effects. This result profoundly reflects the deeply ingrained negative impact (e.g., controlling behaviors, emotional pressure) that highly mathematics education anxious parents may have had on MD children over a long period, forming negative cognitive and emotional patterns that are not easily changed (Oh et al., 2022; Retanal et al., 2021). Therefore, a single or small amount of suggestion may be insufficient to break the MD children’s deep-seated resistance and negative cognition, and the intervention effect may even be greatly reduced because the parents’ own educational anxiety (even when attempting positive suggestions) has not been fundamentally alleviated (Maloney et al., 2015). In this situation, higher frequency and longer duration of positive suggestions are needed to gradually counteract these deep-seated negative effects, rebuild MD children’s learning confidence and positive emotions, and gradually repair potentially damaged parent–child trust relationships. This finding suggests that for MD children tutored by highly mathematics education anxious parents, the formulation of intervention strategies needs to be more cautious and diversified. Relying solely on positive suggestion may not be enough to fundamentally solve the problem, and it may be necessary to combine various methods such as parental psychological counseling and cognitive behavioral therapy to improve their educational effectiveness.

Furthermore, this study also re-emphasized the significant differences in intervention needs between MD children and NMD children. Children with mathematics learning difficulties may have inherent mathematical cognitive deficits or problems with using poor learning strategies (Geary et al., 2012; Geary et al., 2007; Geary et al., 1991). Relying solely on positive suggestion may not be enough to fundamentally address their core learning obstacles, thus requiring the combination of other more targeted intervention methods, such as cognitive skills training, learning strategy guidance, and multi-sensory teaching (Geary et al., 2012; Y. Wang et al., 2022; Q. Zheng & Xu, 2020). Moreover, MD children, due to long-term academic setbacks, may be more susceptible to negative emotions (Gallegos et al., 2012; Y. J. Chen et al., 2014). If the “positive suggestions” from highly mathematics education anxious parents are accompanied by implicit high expectations and pressure, they may instead exacerbate their anxiety, thereby reducing learning effectiveness (J. W. Wang & Wang, 2025; Ma & Zhang, 2022). Indeed, the data suggest this may be precisely the case for non-MD children, whose performance declined under high-frequency suggestions from high-anxiety parents, indicating a clear negative effect. Therefore, for MD children, especially those from highly mathematics education anxious families, the formulation of intervention strategies needs to be more refined and diversified, combining emotional support with cognitive interventions.

These complex findings yield highly specific practical implications that move beyond a generic “be more positive” message. The results suggest that intervention strategies must be tailored to the specific dyad of parent anxiety and child type. For low-anxiety parents with non-MD children, the consistent, high-frequency positive encouragement is a powerful tool to boost achievement. For high-anxiety parents with non-MD children, the primary intervention should target the parents’ anxiety first, before prescribing behavioral changes like “more praise”. Simply asking these parents to provide more positive suggestions may be counterproductive or even harmful, as their underlying anxiety can turn encouragement into pressure. For parents of MD children, the priority is creating a low-anxiety home environment, which acts as a protective buffer. In this context, even modest positive support can be effective. If the environment is already high-anxiety, then a more intensive, high-frequency dose of positive support is necessary to make a tangible difference. In essence, the findings advocate for a shift from a one-size-fits-all approach to a diagnostic, “precision-parenting” model, where the family’s emotional climate dictates the appropriate intervention strategy.

### 4.3. The Drivers of Parental Mathematics Education Anxiety

To develop a more comprehensive understanding of the PMEA phenomenon, the analysis also explored its underlying drivers, revealing that PMEA is not merely an individual psychological issue but is deeply shaped by the broader socio-ecological context.

First and foremost, the results empirically demonstrate that socioeconomic status—encompassing both family income and the tutoring parent’s education level—is the most powerful predictor of PMEA. Parents from lower-income households and those with lower educational attainment reported significantly higher levels of PMEA. This finding strongly supports the notion of ‘socioeconomic imperatives,’ a phenomenon where education is often perceived as the primary, high-stakes pathway for intergenerational mobility. This pressure is intensely felt by both low-income families in developing nations, who make significant financial sacrifices for ‘shadow education’ (Bray & Kwo, 2014; Kim & Jung, 2019), and immigrant families in developed countries (Suárez-Orozco et al., 2008). The anxiety stems directly from the structural pressures of unequal educational resources; lacking the financial means for expensive tutoring or the cultural capital to navigate a competitive system, these parents experience a deeper fear that their children could fall into a cycle of intergenerational poverty (Tian et al., 2024). This suggests PMEA is not merely an individual emotional response but is strongly shaped by structural inequalities.

A secondary, socio-cultural factor is suggested by the finding of a marginally significant increase in parental mathematics anxiety (a related but distinct construct) when the child was a girl. This finding must be interpreted with caution. While this trend did not reach full statistical significance for PMEA itself, its direction points toward the global persistence of gender stereotypes in mathematics (Guiso et al., 2008). Parents’ anxieties may reflect and perpetuate pervasive societal beliefs that girls are less capable in STEM fields, a phenomenon documented across cultures to negatively impact daughters’ confidence and performance (Eccles & Jacobs, 1986; Geary et al., 2023).

In summary, the empirical results prioritize socioeconomic factors as the most significant drivers of PMEA. This underscores that effective solutions must be socially sensitive and address these structural pressures. Governments worldwide have adopted various approaches to mitigate such pressures. For example, contrasting targeted interventions like the Head Start Program in the United States, which provides comprehensive support for low-income families with systemic reforms like China’s nationwide “Double Reduction” (Shuāng Jiǎn) policy, reveals different government approaches to mitigating such pressures. Based on this comparative perspective, the findings recommend that governments prioritize systemic interventions that promote educational equity. Specifically, by narrowing the resource gap between schools through measures like the “balanced development of compulsory education,” it is possible to fundamentally ease the anxiety linked to school selection and the perceived need for costly tutoring, thereby providing universal support that is particularly beneficial for financially strained families.

### 4.4. Research Limitations and Future Outlook

Despite its contributions, this study has several limitations that offer important directions for future research. This study has organized these into three key areas: sample representativeness, methodology, and the theoretical model.

Firstly, the generalizability of the findings is constrained by the nature of the sample. The participants were drawn exclusively from a single primary school in Henan Province, China, resulting in a sample that is relatively homogeneous in ethnicity (predominantly Han Chinese) and limited in geographical and socioeconomic diversity. This restricts the external validity of the conclusions, making it difficult to generalize them to other regions, educational stages (e.g., junior high or high school), or different cultural contexts. Future research should prioritize recruiting more diverse and representative samples from various urban and rural areas, economic backgrounds, and age groups to confirm the broader applicability of the findings.

Secondly, three key methodological limitations warrant discussion, all of which highlight important avenues for future research. The first pertains to instrumentation: the self-developed PMEA scale, while demonstrating good initial content validity and internal consistency, requires full psychometric validation. A critical next step is to establish its factor structure through exploratory and confirmatory factor analysis (EFA/CFA) on larger, independent samples. Subsequent validation should also establish measurement invariance across key demographic groups identified in the study (e.g., parental income and education levels, and child’s gender) and demonstrate convergent and discriminant validity against other established scales. The second relates to the research paradigm. The reliance on quantitative methods, while effective for identifying correlations and intervention effects, provides limited insight into the nuanced, lived experiences within the family context. To capture the dynamic processes of anxiety transmission, future research would be significantly enriched by adopting a mixed-methods paradigm that integrates qualitative approaches, such as in-depth family interviews or diary studies. The third concerns the intervention duration. The 32-day intervention period was sufficient to observe short-term effects but is likely too brief to foster lasting habits, as suggested by research from Lally et al. (2010). Future studies should incorporate longer intervention periods and follow-up tests to assess the long-term stability and sustainability of the observed effects.

Finally, the theoretical model could be further refined. While this study posits that parental anxiety is transmitted to children, this study did not directly measure children’s own mathematics anxiety as a potential mediating variable. This omission is a key limitation. Future research should include such measures to formally test the proposed intergenerational transmission mechanism, which would provide a more granular and complete understanding of the psychological processes at play.

## 5. Conclusions

This study, through two empirical investigations, deeply explored the impact of parental mathematics education anxiety on children’s mathematics achievement and the moderating role of positive suggestion intervention. The following main conclusions were drawn: (1) Parental mathematics education anxiety is a significant negative predictor of children’s mathematics achievement, independent of other forms of anxiety, highlighting the uniqueness and importance of “mathematics education anxiety” as a specific concept; (2) The effectiveness of positive suggestion intervention varies significantly depending on parental mathematics education anxiety levels and child types (MD children/NMD children), suggesting that intervention strategies should be differentiated and individualized. These findings not only provide new evidence for understanding the intergenerational transmission mechanisms of family education anxiety but also offer valuable practical insights for educators and parents, guiding them on how to more precisely improve children’s mathematics achievement.

## Figures and Tables

**Table 1:** Demographic Characteristics of the Participants.

	Category	*n*	Percentage (%)
Parental Role	Father	30	11.54%
	Mother	230	88.46%
Child’s Gender	Male	126	48.46%
	Female	134	51.54%
Single-Child State	Yes	33	12.69%
	No	227	87.31%
Family Structure	Intact family	248	93.38%
	Non-intact family	12	4.62%
Parental Education Level	High school or below	159	61.16%
	College degree or above	101	38.84%
Monthly Family Income	Below 5000 RMB	125	48.08%
	5000 RMB or above	135	51.92%

**Table 2:** Means and Standard Deviations (SD) of Parental Anxiety as a Function of Demographic Variables.

			State Anxiety	Math Anxiety	Math Education Anxiety
Child’s Gender	Male (*n* = 126)	M	37.73	1.72	56.00
		SD	10.871	0.71	13.31
	Female (*n* = 134)	M	38.07	1.96	58.83
		SD	11.22	0.78	14.07
Family Monthly Income	Below 5000 RMB (*n* = 125)	M	39.06	1.94	60.09
		SD	10.62	0.78	13.99
	5000 RMB or above (*n* = 135)	M	36.84	1.75	55.02
		SD	11.33	0.72	13.12
Parental Education Level	High school or below (*n* = 159)	M	38.86	1.91	59.20
		SD	10.31	0.74	13.88
	College degree or above (*n* = 101)	M	36.41	1.74	54.72
		SD	11.98	0.77	13.15

**Table 3:** Correlation analysis of parental state anxiety, mathematics anxiety, and mathematics education anxiety.

	*M*	*SD*	1	2	3	4
1. State Anxiety	37.90	11.30	1			
2. Math Anxiety	1.84	0.75	0.326 ***	1		
3. Math Education Anxiety	57.46	13.75	0.198 ***	0.387 ***	1	
4. Math Achievement	82.85	7.99	−0.089	−0.194 **	−0.333 ***	1

Note: ** *p* < 0.01. *** *p* < 0.001.

**Table 4:** Multiple linear regression analysis with children’s mathematics achievement as the dependent variable.

Variable	B	*SE*	β	*t*
Intercept	94.561	2.375		39.824 ***
state anxiety	−0.003	0.045	−0.004	−0.063
mathematics anxiety	−0.800	0.704	0.075	−1.138
mathematics education anxiety	−0.176	0.037	−0.303	−4.746 ***
R^2^	0.116			
ΔR^2^	0.116			
*F*	11.213 **			

Note: ** *p* < 0.01, *** *p* < 0.001.

**Table 5:** Means (*M*) and Standard Deviations (*SD*) of Pre- and Post-Intervention Mathematics Achievement T-Scores as a Function of PMEA Level, Child Type, and Suggestion Frequency.

PMEA Level	Child Type	Pre-Intervention Achievement *M* (*SD*)	Suggestion Intervention Frequency	Post-Intervention Achievement *M* (*SD*)
Low PMEA	NMD	55.61 (4.49)	Low Suggestion	51.49 (5.51)
			High Suggestion	53.34 (3.64)
	MD	41.44 (4.76)	Low Suggestion	49.63 (6.22)
			High Suggestion	48.00 (6.38)
High PMEA	NMD	52.95 (4.32)	Low Suggestion	50.08 (6.19)
			High Suggestion	48.07 (7.20)
	MD	40.63 (6.93)	Low Suggestion	40.94 (8.43)
			High Suggestion	49.48 (4.19)

## Data Availability

Dataset available on request from the authors.

## Appendix A

**Table A1:** Parental Mathematics Education Anxiety (PMEA) Questionnaire.

1. When doing math homework, the child always likes to procrastinate and dawdle.	1	2	3	4	5
2. The child’s math scores are always lower than other subjects.	1	2	3	4	5
3. The child always wants to copy answers when doing math problems.	1	2	3	4	5
4. Other people’s children are better at math than my child.	1	2	3	4	5
5. No matter how much effort is put in, the child’s math performance only declines, not improves.	1	2	3	4	5
6. If the child is not good at math now, it will be difficult to find a job in the future.	1	2	3	4	5
7. I never enroll the child in various math tutoring classes.	1	2	3	4	5
8. I buy more math materials for the child than for other subjects.	1	2	3	4	5
9. I spend more time tutoring the child in math homework than in other subjects.	1	2	3	4	5
10. If the child is poor at math now, they will be even worse in higher grades.	1	2	3	4	5
11. Seeing the child’s homework and test papers full of mistakes makes me furious.	1	2	3	4	5
12. Every time I think about tutoring the child in math, I get a headache.	1	2	3	4	5
13. I worry a lot about the child’s math learning.	1	2	3	4	5
14. For this item, please choose “Strongly Agree”.	1	2	3	4	5
15. * I never buy extra-curricular tutoring materials for the child.	1	2	3	4	5
16. The child is not good at math now, and I worry about future college entrance exams.	1	2	3	4	5
17. The child always learns math by rote.	1	2	3	4	5
18. The math teacher reports that the child does not listen in class.	1	2	3	4	5
19. The child is always careless and does not read the questions carefully.	1	2	3	4	5
20. Because I am not good at math myself, I cannot provide better guidance to the child.	1	2	3	4	5
21. When tutoring the child in math, I always lose my temper.	1	2	3	4	5

Please rate the following statements on a 5-point Likert scale, where 1 = Strongly Disagree and 5 = Strongly Agree. (Note: * Items are reverse-scored).
